# Targeting hepatic macrophages for non-alcoholic fatty liver disease therapy

**DOI:** 10.3389/fcell.2024.1444198

**Published:** 2024-09-05

**Authors:** Yingxin Tian, Yiming Ni, Ting Zhang, Yemin Cao, Mingmei Zhou, Cheng Zhao

**Affiliations:** ^1^ Shanghai Traditional Chinese Medicine Integrated Hospital, Shanghai University of Traditional Chinese Medicine, Shanghai, China; ^2^ Institute of Interdisciplinary Integrative Medicine Research, Shanghai University of Traditional Chinese Medicine, Shanghai, China; ^3^ School of Pharmacy, Shanghai University of Traditional Chinese Medicine, Shanghai, China

**Keywords:** non-alcoholic fatty liver disease (NAFLD), macrophages, Kupffer cells (KCs), monocyte-derived macrophages (MoMFs), NASH

## Abstract

Non-alcoholic fatty liver disease (NAFLD) and its more advanced form, non-alcoholic steatohepatitis (NASH), have become global health challenges with significant morbidity and mortality rates. NAFLD encompasses several liver diseases, ranging from simple steatosis to more severe inflammatory and fibrotic forms. Ultimately, this can lead to liver cirrhosis and hepatocellular carcinoma. The intricate role of hepatic macrophages, particularly Kupffer cells (KCs) and monocyte-derived macrophages (MoMFs), in the pathogenesis of NAFLD and NASH, has received increasing attention. Hepatic macrophages can interact with hepatocytes, hepatic stellate cells, and endothelial cells, playing a crucial role in maintaining homeostasis. Paradoxically, they also participate in the pathogenesis of some liver diseases. This review highlights the fundamental role of hepatic macrophages in the pathogenesis of NAFLD and NASH, emphasizing their plasticity and contribution to inflammation and fibrosis, and hopes to provide ideas for subsequent experimental research and clinical treatment.

## 1 Introduction

Non-alcoholic fatty liver disease (NAFLD) is recognized worldwide as a rapidly evolving manifestation of the metabolic syndrome (Quek et al., 2023). Non-alcoholic steatohepatitis (NASH) is an evolving form of NAFLD characterized by excessive triglyceride accumulation (steatosis), inflammation, injury, and hepatocyte apoptosis leading to fibrosis, cirrhosis, and hepatocellular carcinoma. The progression of NASH is currently a major cause of liver transplantation (Younossi et al., 2018). In 2023, three major multinational liver associations recommended substituting the term NAFLD with metabolic dysfunction-associated steatotic liver disease (MASLD) and replacing NASH with metabolic dysfunction-associated steatohepatitis (Rinella et al., 2023; Targher et al., 2024). Recent epidemiological data indicates a high degree of agreement between NAFLD and MASLD definitions, with approximately 99% of NAFLD cases meeting MASLD criteria (Hagström et al., 2024). Nevertheless, MASLD’s greater emphasis on the complex relationship between hepatic fat deposition and metabolic dysfunction results in some differences between MASLD and NAFLD (He et al., 2024). As there is still limited experimental evidence and research to validate the definition and diagnostic criteria of MASLD, this paper continues to use the definitions of NAFLD and NASH for the overview (Cusi et al., 2024).

The important role of hepatic macrophages in the development and recovery of NAFLD and NASH has been well documented (Rinella et al., 2023; Targher et al., 2024). Hepatic macrophages incorporate Kupffer cells (KCs), which are derived embryonically, and circulating monocyte-derived macrophages (MoMFs) (Tacke and Zimmermann, 2014). As an essential cellular constituent of the liver, hepatic macrophages are central in promoting repairment after hepatic injury and regulating tissue homeostasis in physiological conditions and pathology (Igarashi et al., 2024). From early animal models and human clinical trials, it can be reasonably concluded that targeted pathogenic hepatic macrophages offer a promising avenue for the treatment of NAFLD and NASH (Xu et al., 2022).

This review will focus on recent developments in hepatic macrophages in NAFLD and NASH and their potential for innovative treatment approaches.

## 2 Pathogenesis of NAFLD/NASH

NAFLD is a multifaceted disorder encompassing a wide range of liver conditions. The spectrum of NAFLD ranges from the accumulation of fat in over 5% of hepatocytes (simple steatosis) to NASH, which is distinguished by the presence of necro-inflammation, hepatocyte injury, and progressive fibrosis (Friedman et al., 2018). In physiological states, lipids play crucial roles in intracellular metabolism, intercellular signaling, the regulation of inflammation, and the structural integrity of membranes. In hepatocytes, triglycerides are typically stored properly as lipid droplets to mitigate lipotoxicity by reducing extracellular free fatty acids (FFAs). Conversely, erroneous deposition or mobilization of lipid droplets and alterations in protein expression can exacerbate hepatocyte lipotoxicity, thereby contributing to the progression of NAFLD (Yamaguchi et al., 2007; Su et al., 2014). When the uptake and synthesis of fatty acids exceed the rate at which they are eliminated, it initiates cellular stress and lipid toxicity. This, in turn, causes cellular damage by disrupting the function and structure of intracellular organelles such as the endoplasmic reticulum and mitochondria. Additionally, it interferes with intracellular pathways and interacts with specific pro-inflammatory cell kinases, which can be located on the cell surface or within the cytoplasm. Ultimately, these are responsible for the development and progression of NAFLD to NASH (Moayedfard et al., 2022).

## 3 Main components of hepatic macrophages in NAFLD

The liver contains two major classes of macrophages: resident macrophages, also known as KCs, and MoMFs (Hagström et al., 2024). Both KCs and MoMFs can play a role in both the development and repair of inflammation and injury in hepatic disease.

### 3.1 KCs

KCs represent approximately 15% of the total liver cell population. They account for 80%–90% of the total macrophage population in the body and are essential for maintaining hepatic tolerance. A high abundance of KCs indicates their crucial role in maintaining hepatic function and balancing the microenvironment (Han M. et al., 2023).

While both KCs and recruited macrophages express some common markers, they also exhibit distinct surface proteins that allow for their differentiation. KCs, which are resident macrophages in the liver, express high levels of scavenger receptors such as scavenger receptor A and MARCO. These receptors are involved in the recognition and clearance of modified lipoproteins and pathogens (Li et al., 2014). Furthermore, they express Fcγ receptors (e.g., CD64) (Rolot et al., 2019) for antibody-mediated phagocytosis, and T-cell immunoglobulin domain and mucin domain-4 (TIM-4), which is involved in the binding and clearance of apoptotic cells (Ni et al., 2021). Additionally, KCs are characterized by the expression of C-X3-C motif receptor 1, a chemokine receptor linked to patrolling functions (Wang J. et al., 2020). In contrast, recruited macrophages frequently express CC-chemokine receptor-2 (CCR2), a chemokine receptor that is essential for the migration of monocytes from the bone marrow to sites of inflammation (Mulder et al., 2017). These cells also express the receptor for macrophage colony-stimulating factor, which regulates their proliferation and survival (Stadler et al., 2021). Other markers include CD14 (Roedig et al., 2019), which is involved in the response to bacterial lipopolysaccharide, and CD163 (Xi et al., 2021), a hemoglobin scavenger receptor linked to iron metabolism and anti-inflammatory functions (Guo et al., 2018).

Specific surface markers, such as F4/80hi (mouse) and CD11bint (human), are expressed by KCs, facilitating their identification. Mouse Kupffer cells (KCs) are hepatic macrophages that specifically express C-type lectin domain family 4 member F, irrespective of their origin (Scott et al., 2016). Additionally, C-type lectin domain family 2 (Tran et al., 2020), TIM4 (Ni et al., 2021), and V-set and immunoglobulin domain-containing 4 (Guilliams et al., 2022) are additional surface markers of mouse KCs. While human KCs do not express a direct equivalent of F4/80^hi^, but they express CD14, a co-receptor for lipopolysaccharide recognition (Ikarashi et al., 2013). CD163, a hemoglobin scavenger receptor, is highly expressed in human Kupffer cells and is involved in iron metabolism and anti-inflammatory functions (Li X. et al., 2022). Both mouse and human KCs express CD11b and TIM-4 (Wu et al., 2020; Ni et al., 2021).

Additionally, high densities of scavenger receptors (Zhang et al., 2019), complement receptors (Sun et al., 2019), Toll-like receptors (Labiano et al., 2022), Nod-like receptors (Yu et al., 2017), and mannose receptors (Maeda et al., 2022) are also enriched in the surface of KCs. Furthermore, KCs are capable of secreting cytokines that regulate the inflammatory response and other bioactive molecules through pattern recognition receptors (Yao et al., 2023).

#### 3.1.1 The sources of KCs

Yona et al. proposed that hepatic KCs are formed before birth and maintained in adulthood through self-renewal. During the early stages of hepatic injury, the number of KCs decreases, and subsequently, they are replenished through their proliferation, with infiltrating macrophages not participating in the replenishment of KCs during the injury process (Yona et al., 2013).

However, recent research indicates that the origin of hepatic resident macrophages, including KCs, may be more complex and diverse than previously thought. During the early stages of hepatic injury, the population of KCs declines, yet they are subsequently replenished through proliferation to restore the KC pool (Li et al., 2023). In addition, in certain circumstances, hepatic resident macrophages, including KCs, may originate from both embryonic precursor cells and other exogenous cell populations (Hoeffel et al., 2015). For instance, peripheral blood monocytes and monocytes in the bone marrow can enter the liver through the circulatory system and differentiate into hepatic resident macrophages (Tran et al., 2020; Wang and Gao, 2021).

In NASH mice, monocytes (TIM^neg^ macrophages) also contribute to the replenishment of the KCs pool. The results of immunolabeling studies have demonstrated that monocyte-derived KCs are present in the hepatic sinusoids, in a manner analogous to that observed in embryonically derived KCs. The production of monocyte-derived KCs in NASH patients is a response to increased embryonically derived macrophage death and aims to maintain the population of KCs (Seidman et al., 2020; Daemen et al., 2021).

#### 3.1.2 The activation of KCs in NAFLD and NASH

Elevated levels of FFAs and triglycerides can result in increased lipid oxidation, reactive oxygen species generation, and lipotoxicity, collectively contributing to cellular injury. This, in turn, can lead to the release of cytokines that trigger the activation of KCs and attract monocytes and other immune cells from the bloodstream (Xu G.-X. et al., 2023).

**FIGURE 1:**
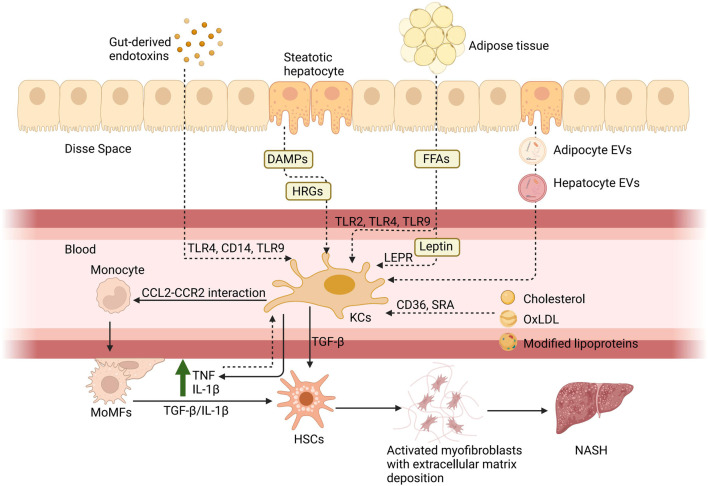
Hepatic macrophages in NAFLD. There are six principal pathways by which KCs are involved in the pathogenesis of NAFLD: 1) the entry of gut-derived endotoxins into the circulation due to enhanced intestinal permeability and their binding with toll-like receptors; 2) via molecules associated with hepatocyte damage such as histidine-rich glycoprotein (HRG) and DAMPs; 3) via FFAs acting through TLRs and adipokines such as leptin binding with the leptin receptor (LEPR) from adipose tissue; 4) via cholesterol and its metabolites, including oxidized low-density lipoprotein (oxLDL), which act through CD36 and scavenger receptor A; 5) via the direct action of adipocyte extracellular vesicles (EVs) and hepatocyte EVs to KCs; 6) and the rise of TNF and IL-10 caused by inflammation after the activation can also activate KCs. Subsequently, KCs will release pro-inflammatory cytokines, such as TNF and IL-1β, to assist monocytes through the CCL2-CCR2 interaction. Monocytes differentiate into MoMFs to exacerbate hepatic inflammation and contribute to fibrosis through stimulation of HSCs by TGF-β and IL-1β. Created with BioRender.com.

**FIGURE 2:**
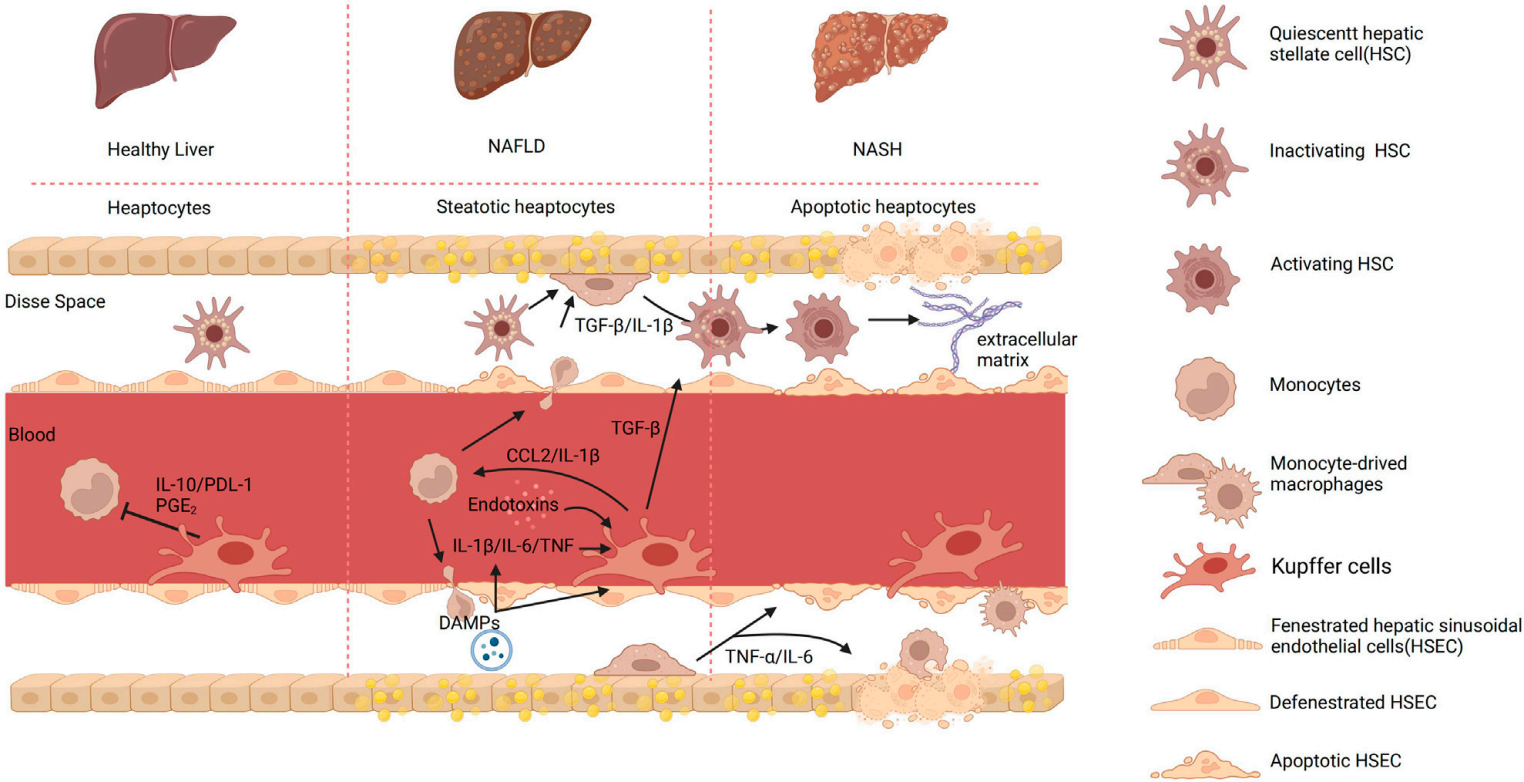
The process of KCs and cell-to-cell signaling in different hepatic environments. In a healthy organism, there exists intercellular communication between monocytes and KCs mediated by IL-1β and programmed cell death ligand 1 (PDL-1). This communication regulates inflammation and immune responses. IL-10 is an anti-inflammatory cytokine produced by monocytes, which acts as a signaling molecule to communicate with KCs. It inhibits the production and release of various inflammatory cytokines, thereby reducing inflammation. In contrast, KCs employ the surface molecule PDL-1 to modulate T-cell activation, thereby regulating immune responses and restraining excessive inflammatory reactions. Furthermore, prostaglandin E2 (PGE2) can be produced by both monocytes and KCs and acts as a signaling molecule that affects the immune response. PGE2 can influence the function of KCs and monocytes by regulating their activation, cytokine production, and migration. Upon detecting abnormalities in the body, KCs become activated and transmit signals to activate HSCs, which in turn promote the phagocytosis and apoptosis of damaged cells. Monocytes can also infiltrate the Disse space, where they participate in inflammatory responses and tissue repair. The interaction between endotoxins and KCs can trigger an immune inflammatory response. KCs recognize endotoxins and release pro-inflammatory cytokines, which lead to liver inflammation. In addition, in NAFLD, damaged liver cells release signaling molecules known as DAMPs. The release of DAMPs can activate KCs, leading to the secretion of pro-inflammatory cytokines such as TNF-α and IL-1β. Both KCs and macrophage-derived cytokines can induce the activation of HSCs and the deposition of extracellular matrix, leading to the formation of fibrous septa. Created with BioRender.com.

After KCs activation, the participation of KCs, and MoMFs in NAFLD is shown in Figure 1. This cascade then leads to the apoptosis of the hepatocytes and the subsequent mobilization of the hepatic stellate cells (HSCs). Ultimately, it causes the deposition of the extracellular matrix, which, if excessive, can lead to fibrosis (Cao et al., 2022). The progression of KCs in NAFLD and cell-to-cell signaling is shown in Figure 2.

Given the association between the functional activity of KCs and the development of pathological states, the inability of KCs to regulate the inflammatory response with precision would result in hepatic chronic inflammation, including NAFLD and NASH, by increasing the expression of proinflammatory factors and genes related to fibrosis and oxidative damage (Dixon et al., 2013; Chen et al., 2020). During chronic inflammation, KCs undergo a transition from a tolerogenic phenotype to a pathologically activated status, causing liver injury (Chen et al., 2020). A wealth of clinical evidence and experimental data has demonstrated that KC activation is a crucial initial step in hepatic injury (Lucey et al., 2009; Sakaguchi et al., 2011; Wan et al., 2014). The subsequent enhanced release of M1 mediators derived from KCs will then lead to the pathogenesis of various liver lesions, including steatosis, apoptosis, inflammatory cell recruitment, and fibrosis (Reid et al., 2016; Zhang Y. et al., 2023).

The gut microbiota, a diverse community of microorganisms in the digestive tract, has been demonstrated to profoundly impact human health, including immunity, metabolism, and inflammation. Recent findings suggest that it affects KCs, hepatic cells’ function, and behavior. Microbes’ components absorbed from the intestines influence the liver, forming a “gut-liver axis” (Kamiya and Ohtani, 2022). Gut bacteria-derived lipopolysaccharides (LPS) can enter the liver through the portal vein, activating KCs’ immune responses. This results in the production of inflammatory cytokines and the initiation of hepatic inflammation (Cani et al., 2007; Siebler et al., 2008). Gut microbiota-generated metabolites, such as short-chain fatty acids, which are derived from the fermentation of dietary fiber, have anti-inflammatory effects. They influence the activation of KCs, promoting an anti-inflammatory role (Beaumont et al., 2018). According to the pathological processes of NAFLD, this article here divides the pathological processes of NAFLD into three parts steatosis, inflammation, and fibrosis (Friedman et al., 2018; Chen et al., 2020).

#### 3.1.3 KCs in the progression of hepatic steatosis in NAFLD

Hepatic steatosis represents the initial stage of NAFLD. Initial research has indicated that decreased hepatic enhancement is positively correlated with the attenuated phagocytic function of KCs as evidenced by superparamagnetic iron oxide magnetic resonance imaging of patients with NAFLD. This research addresses a significant gap in our understanding of the pathogenesis of human NAFLD, as animal models cannot fully reflect the disease (Asanuma et al., 2010). The development of NASH is mediated by KCs and HSCs and relates to congenital immunity. Toll-like receptor 9 (TLR9) is a pattern-recognizing receptor that recognizes cytosine-phosphate-guanine from bacteria, activating the innate immune system. Previous research has shown that TLR9 signaling promotes KC production of interleukin-1β (IL-1β) in a mouse model of NASH, contributing to steatosis, inflammation, and fibrosis (Shepard, 2020). It is noteworthy that the inhibition of TLR-9 expression in mouse models fed a high-fat diet has been demonstrated to result in a reduction in the NASH phenotype and a significant shift towards the M2 phenotype in KCs, accompanied by a notable control of M1 activation (Mridha et al., 2017a). These experiments shed light on the mechanism by which KCs activation in NAFLD leads to steatosis and hepatic damage.

The degree of hepatic steatosis is influenced by the cumulant of triglycerides, and the accumulation of other lipids such as phospholipid and cholesterol in the liver could also cause lipotoxicity and proinflammatory factor secretion of KCs (Manne et al., 2018). Excessive cellular lipid accumulation inevitably activates hepatic macrophages, promoting the formation and progression of NASH (Zhang et al., 2024). Consequently, a potential therapeutic approach is to prevent the accumulation of lipotoxic lipids in the liver. Concerning dietary intervention, lycopene has been demonstrated to inhibit the development of hepatic steatosis induced by a high-fat diet. Conversely, a low-lycopene condition may result in the progression of NAFLD (Bahcecioglu et al., 2010; Erhardt et al., 2011). Additionally, β-carotene, astaxanthin, and β-cryptoxanthin were demonstrated to exhibit analogous effects (Kobori et al., 2014; Ni et al., 2015; Yilmaz et al., 2015). It is of particular note that astaxanthin represents a promising therapeutic option for NAFLD. This is due to its capacity to inhibit lipid accumulation and proinflammatory signaling, as well as its ability to act directly on KCs to promote the activation of M2 macrophages (Ni et al., 2015).

Glucose is converted into pyruvate through glycolysis in the cytoplasm, and pyruvate is subsequently oxidized in the mitochondria to generate ATP through the tricarboxylic acid cycle and oxidative phosphorylation. In the fed state, glycolytic products are utilized for the synthesis of fatty acids through *de novo* lipogenesis (Rui, 2014). G protein-coupled receptors play a pivotal role in the pathogenesis of metabolic disorders, functioning as receptors for metabolites and fatty acids. It has been demonstrated that the activation of G-protein coupled receptor 3 in KCs stimulates glycolysis and protects mice from obesity and fatty liver disease. This process involves the interaction of G-protein coupled receptor 3 with β-arrestin2, pyruvate kinase M2, and the c-Myc pathway, which plays a crucial role in metabolic reprogramming in macrophages. The activation of this pathway in KCs represents a promising approach for the treatment of NAFLD (Dong et al., 2024).

#### 3.1.4 KCs in the inflammatory process of NAFLD

In NAFLD, KCs can be activated by a variety of stimuli, including lipids and inflammatory cytokines. This activation leads to the secretion of pro-inflammatory mediators and the initiation of an inflammatory response (Maeda et al., 2022). Normally, triglyceride deposits in hepatic cells in the form of lipid droplets serve to mitigate lipotoxicity and hold back the progression of NAFLD. For many years, it has long been widely accepted that inflammation represents a pivotal link between NAFLD and NASH. TLRs are cytoplasmic pattern recognition receptors with potent inflammatory effects in hepatic cells. Upon sensing their corresponding ligands and metabolites, TLRs can exacerbate local inflammation (Baumann et al., 2021).

There are several mechanisms by which KCs contribute to the progression of NAFLD. Firstly, they release pro-inflammatory cytokines, including the nucleotide-binding oligomerization domain, leucine-rich repeat-containing receptor (NLR) family pyrin domain-containing 3 (NLRP3), tumor necrosis factor-α (TNF-α), IL-1β, IL-6, IL-10, IL-12, IL-18, which promote hepatic inflammation and injury after intestinal LPS reaches the liver. These cytokines stimulate the recruitment and activation of other immune cells, which can exacerbate the inflammatory response (Tacke, 2017; Maeda et al., 2022). Inflammasomes, including NLRP-1, NLRP-3, NLRP-4, and absent in melanoma 2, recognize endogenous or exogenous pathogen-associated molecular patterns or danger-associated molecular patterns (DAMPs) and induce the release of inflammatory cytokines, such as pro-IL-1β and pro-IL-18 (Liu et al., 2023). The NLRP3 inflammasome is a crucial factor in processing key pro-inflammatory cytokines, making it a potential target for the prevention and treatment of NAFLD/NASH (Cao et al., 2022). In recent years, numerous studies using NLRP3 inflammasome-related drugs and inhibitors in experimental models have supported the therapeutic approach for treating NAFLD/NASH, demonstrating positive effects. Translating these findings into therapeutic support may represent a novel approach to treating inflammatory hepatic diseases (Wang et al., 2022; Ma et al., 2023). The researchers also identified MCC950 as a highly effective and selective NLRP3 inhibitor, demonstrating the ability to mitigate hepatic injury and fibrotic progression of NASH by inhibiting NLRP3 activation (Mridha et al., 2017b). However, due to the lack of clarity regarding the oral dose of MCC950, the full potential of inflammasome inhibition remains untapped. In contrast to MCC950, CY-09, a novel selective and direct NLRP3 inhibitor, has the potential to treat NAFLD and NASH by inhibiting weight gain and visceral lipid deposition in NAFLD mice. While the mode of action differs from that of MCC950 and requires further investigation, CY-09 represents a promising avenue for the treatment of NAFLD and NASH (Wang et al., 2021). A study conducted several years ago revealed that the Yes-associated protein in KCs has the capacity to enhance the generation of pro-inflammatory factors and facilitate the development of NASH (Song et al., 2020).

Secondly, activated KCs can release reactive oxygen species to circumambient hepatocytes and stellate cells by the activation of NADPH oxidase 2 and TLR signaling pathways, leading to oxidative stress and cellular damage. The increased oxidative stress contributes to hepatocyte injury and promotes the progression of hepatic inflammation in NAFLD (Maeda et al., 2022; Zhang et al., 2024).

KCs are also responsible for producing chemokines and adhesion molecules that are critical for recruiting inflammatory cells into the liver. For instance, chemokines such as CC-chemokine ligand 2 (CCL2) and chemokines including chemokine ligand 10 are produced by KCs and facilitate the recruitment of monocytes and T cells to the site of inflammation in the liver. Adhesion molecules, including ICAM-1 and VCAM-1, are expressed on the surface of KCs, which enables the adhesion and migration of inflammatory cells into liver tissue. The recruitment and activation of inflammatory cells contribute to the perpetuation of hepatic inflammation in conditions such as NAFLD (Tacke, 2017).

#### 3.1.5 KCs in the fibrosis of NAFLD

The intensification of inflammation and fibrosis is a consequence of the actions of HSCs, which produce excessive extracellular matrix and inflammatory mediators. This is due to the activation of these cells that is caused by apoptotic hepatocytes and KCs (Cao et al., 2022; Liu et al., 2023). TLR-2, TLR-4, and TLR-9, which are predominantly expressed in KCs, are critical in the pathogenesis of steatosis, inflammation, and fibrosis in several diet-induced mouse models of NAFLD and NASH (Miura et al., 2010; Nakamoto and Kanai, 2014; Yin et al., 2014). In contrast, TLR-5 has been shown to exert an opposing effect, namely, by resisting the entry of circulating gut bacteria and high-fat diet-induced steatosis and fibrosis (Etienne-Mesmin et al., 2016). Despite the considerable functions of TLRs, extensive activation or suppression of inflammatory signals appears to be an unfeasible therapeutic approach. Recently, 44 miRNAs were identified as being abnormally expressed in NAFLD livers in comparison to normal livers. This discovery has led to research investigating the pathomechanism of NAFLD and NASH through miRNA analysis (Ying et al., 2017; Gjorgjieva et al., 2019; Mahmoudi et al., 2022). The majority of miRNAs are secreted from cells via microvesicles as a result of active, energy-consuming processes. In NAFLD, damaged and dead cells shed circulating miRNAs, which may reflect molecular events in the liver (Mahmoudi et al., 2022). In addition, susceptibility to fibrosis in NAFLD can be predicted using miRNA-based diagnostic tools. Although the accurate and detailed diagnosis and treatment of NAFLD and hepatic fibrosis are still in the early stages of clinical trials, there is a great potential for targeting miRNA-based treatment to realize personalized remedies and management for patients with NAFLD and hepatic fibrosis.

The expression of N6-methyladenosine methyltransferase (METTL14) was shown to be significantly diminished in the livers of both patient samples and multiple murine models of MAFLD. Hepatocyte-specific depletion of this methyltransferase was observed to exacerbate lipid accumulation, liver injury, and fibrosis. METTL14 downregulation resulted in a reduction in the level of GLS2, which was found to be mediated by YTHDF1 and dependent on m6A modification. This may result in the formation of an oxidative stress microenvironment, which in turn facilitates the recruitment of Cx3cr1^+^Ccr2^+^ MoMfs. Specifically, these activated MoMfs express S100A4, which further activates HSCs to promote liver fibrosis. Further experiments demonstrated that CX3CR1 can activate the transcription of S100A4 via the CX3CR1/MyD88/NF-κB signaling pathway in Cx3cr1^+^Ccr2^+^ MoMfs (Wang et al., 2024).

In conclusion, KCs exhibit a spectrum of functions in NAFLD and NASH, and their phenotype is influenced by the local metabolic and immune environment. They can adopt a range of polarized phenotypes, from a pro-inflammatory M1 type to an M2 type (Reid et al., 2016). These findings offer new insights into the potential of manipulating KCs to promote inflammation resolution and wound healing in hepatic disorders.

### 3.2 MoMFs

MoMFs represent a discrete subset of macrophages that exhibit remarkable plasticity and functional diversity. These cells are capable of undergoing a phenotypic switch between M1-like and M2-like phenotypes, contingent upon the surrounding milieu. This phenotypic plasticity is accompanied by corresponding functional and phenotypic changes. In the context of NAFLD, the activation and polarization of MoMFs play a crucial role in inflammatory responses, fibrosis processes, and pathological progression (Li et al., 2020).

#### 3.2.1 Polarization and phenotypic transitions of MoMFs in NAFLD

In the early stages of NAFLD, MoMFs exhibit characteristics of M1-like macrophages, releasing inflammatory cytokines and chemokines that contribute to hepatic inflammation and cellular damage. As the disease progresses, MoMFs may transition into M2-like macrophages, displaying anti-inflammatory and reparative properties, and participating in tissue repair and fibrosis processes (Keirsse et al., 2018). For example, transforming growth factor-β (TGF-β) is known to induce the polarization of MoMFs towards the M2-like phenotype, while cytokines including IL-4 and IL-13 also have an impact on M2 polarization (Rahal et al., 2018; Liu et al., 2019; Yang B. et al., 2022; Vadevoo et al., 2024).

The balance between the regulation of activation and polarization of MoMFs may have a critical role in the development and progression of NAFLD. Excessive M1 polarization may contribute to liver inflammation and disease progression through the release of pro-inflammatory cytokines. Conversely, a shift towards M2 polarization and the secretion of anti-inflammatory and reparative factors may promote tissue repair and attenuate inflammation (Wen et al., 2021). To maintain tissue homeostasis and prevent excessive inflammation in NAFLD, it is essential to achieve a balanced polarization state of MoMFs, wherein both the M1 and M2 populations are appropriately regulated.

#### 3.2.2 Chemokine produced by MoMFs in the pathogenesis of NAFLD and NASH

The regulation of monocyte flux to the liver is primarily controlled by the chemokine CCL2, which can interact with its homologous receptor, CCR2, to mediate the migration of monocytes from the bone marrow to inflammatory sites. It has been demonstrated that the expression of CCL2 and CCR2 is inversely correlated in proinflammatory and anti-inflammatory MoMFs. This indicates that the CCL2-CCR2 pair influences macrophage polarization at the transcriptomic and functional levels in human and murine macrophages (Sierra-Filardi et al., 2014).

Despite the lack of understanding surrounding the mechanisms underlying the NAFLD-to-NASH transition, the identification of novel molecules related to this process could help elucidate its underlying molecular mechanisms and facilitate the development of potential therapeutic avenues and strategies. A recent study demonstrated that plasma Sparcl1 levels were positively correlated with pathological features of the liver, including steatosis, ballooning, and lobular inflammation. Sparcl1 levels were significantly higher in patients with NASH compared to those with NAFLD. This suggests that Sparcl1 has the potential to accurately identify NASH in NAFLD patients. Moreover, Sparcl1 can induce and regulate CCL2 to activate an inflammatory response, filling the gap in the field of glycoproteins in NASH (Liu B. et al., 2021). CD44 provides new insights and research avenues into the pathogenesis of NAFLD and NASH. In addition to accommodating the infiltration of monocytes and macrophages into the liver, CD44 deficiency can modify the characteristics of macrophages, rendering them more susceptible to anti-inflammatory M2 polarization and less susceptible to M1 upon activation. The functions of these novel molecules require further investigation and could be a potential therapeutic strategy for correcting NAFLD and NASH.

Macrophage accumulation in fat tissue is an important factor in inflammation associated with obesity and contributes to insulin resistance and fatty liver. A guess that it is higher CCL2 levels in obese patients that cause insulin resistance instead of the accumulation of macrophages has been proved to be correct by some relevant experiments (Tateya et al., 2010; Marra and Tacke, 2014). Additionally, CCR2 plays a vital role in recruiting immune cells to white adipose tissue and hepatic cells, thereby promoting inflammation in NASH. The CCL2/CCR2 axis is critical for the recruitment of monocytes and macrophages, which are associated with several aspects of hepatic disease (Moragrega et al., 2023). Cenicriviroc is an oral dual chemokine receptor CCR2/CCR5 antagonist that is currently under clinical evaluation. It has been demonstrated to inhibit CCR2^+^ monocyte recruitment and improve insulin resistance, hepatic inflammation, and fibrosis. This treatment strategy, which involves initiating therapeutic intervention for NASH at an early stage, has been demonstrated to be effective (Mulder et al., 2017; Tacke, 2017).

In NASH and liver fibrosis, both CCR2 and CCR5 are targets. However, it is possible that targeting CCR2 is more effective than targeting CCL2 because this receptor also binds the ligands CCL7, CCL8, and CCL13 (Lefere et al., 2020).

#### 3.2.3 Interaction between MoMFs and other hepatic cells in NAFLD and NASH

Infiltrative MoMFs are divided into two major subpopulations: pro-inflammatory Ly-6C^hi^ macrophages and pro-resolving Ly-6C^lo^ macrophages. Previous research has shown that MoMFs activate HSCs by secreting TGF-β, IL-1β, and CCL2, thereby amplifying the fibrotic response (Ramachandran et al., 2019). However, recent studies have indicated that in the presence of other soluble factors, the primacy of TGF-β in promoting fibrosis is reduced, suggesting that TGF-β is not the sole factor driving HSC activation (Sauer et al., 2024). On the other hand, pro-resolving Ly-6C^lo^ macrophages facilitate HSCs apoptosis and expedite extracellular matrix degradation by upregulating the expression of matrix metalloproteinase 9 (MMP9), MMP12, MMP13, and insulin-like growth factor 1 (Li et al., 2020). Future research could investigate the specific signaling pathways and molecular mechanisms underlying the pro-inflammatory and pro-resolving functions of MoMFs.

MoMFs were found to be intimately associated with a broad range of T-cell populations in the development of NAFLD, through their secretion of interleukins IL-6, IL-1β, IL-12, IL-23 (Van Herck et al., 2019). In the context of NAFLD, T helper 17 (Th17) cells and their secretion of IL-17 played a pivotal role in advancing the progression from mere steatosis to steatohepatitis. They promoted an enhanced activation of monocytes, triggering a cascade of pro-inflammatory cytokine releases, which consequently intensified hepatic inflammation (Guo et al., 2024). Both innate and adaptive immunity contribute to the progression of NASH. T-cell activation requires antigen presentation by professional antigen-presenting cells, including liver macrophages, dendritic cells, and B cells. Among these, CD4^+^ T cells play a crucial role in the pathogenesis and progression of NASH. Different subsets of CD4^+^ T cells have varying effects on liver fibrosis (Zhou et al., 2022). Th1 and Th17 cells exert pro-inflammatory and pro-fibrotic effects, while the role of Th2 cells in liver fibrosis is more complex. Th22 and Treg cells may have dual roles in anti-fibrotic and/or pro-fibrotic effects, depending on the disease environment and stage (Van Herck et al., 2019; Zhou et al., 2022). Further insights into the immune dysregulation in NAFLD/NASH progression may be gained by exploring the crosstalk between MoMFs and other immune cell populations, such as natural killer cells and regulatory T-cells.

In the early stages of NAFLD, hepatic sinusoidal endothelial cells lose their ability to produce vasodilators, leading to increased hepatic angiogenesis and inflammation. In the NASH stage, liver sinusoidal endothelial cells exhibit an enhanced pro-inflammatory phenotype, characterized by increased production of the chemokine CCL2, which promotes the recruitment of monocytes to the liver (Miyao et al., 2015; Gao et al., 2021). Understanding the dynamic changes in LSEC phenotype and function throughout disease progression could unveil novel therapeutic targets for intervention.

It is noteworthy that the aforementioned interactions between MoMFs and other hepatic cells are predominantly observed in mouse models and mediated through cytokine signaling. The core intracellular pathways and complex cellular networks through which MoMFs mediate intercellular signaling in human NAFLD and NASH remain unclear.

## 4 Hepatic macrophage polarization in NAFLD

**FIGURE 3:**
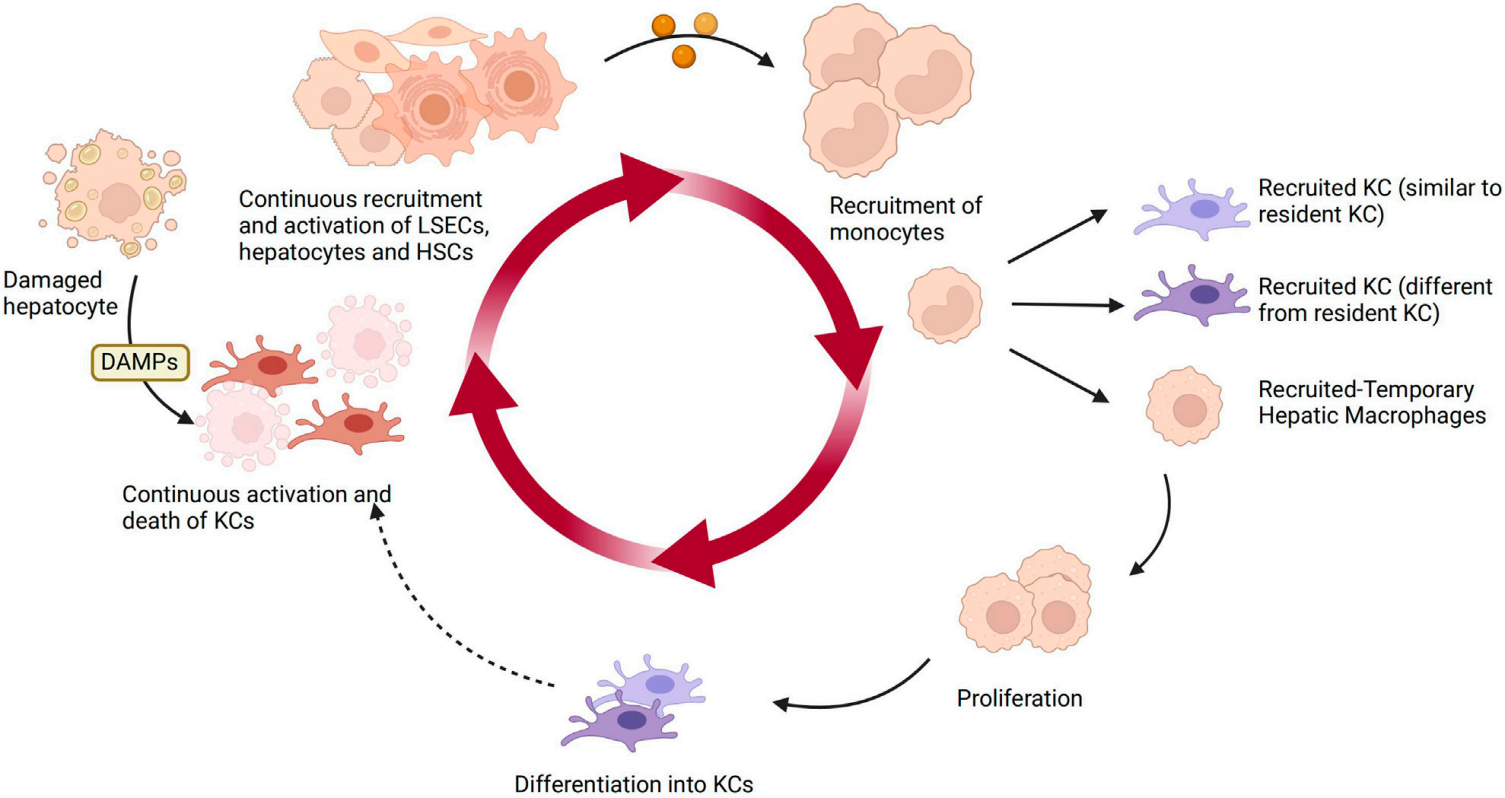
The plasticity of KCs and other hepatic cells in NAFLD. KCs are the main macrophage population in the liver under homeostasis. Upon sensing DAMPs from injured hepatocytes, KCs become activated and produce chemokines and cytokines, recruiting and activating liver sinusoidal endothelial cells, HSCs, and hepatocytes. They also recruit monocytes to replenish the lost KCs in a continuous manner. The recruited monocytes locally differentiate into KCs or temporarily recruited macrophages. Over time, the recruited KCs can continue to circulate. Created with BioRender.com.

Under homeostatic conditions, KCs are the main macrophage population in the liver, primarily responsible for clearing apoptotic cells and microbes and maintaining immune homeostasis. While activated macrophages differentiate or polarize into two distinct subgroups, namely M1 and M2. KCs exhibit remarkable plasticity, as they can adapt their phenotype based on local signals within the hepatic microenvironment. Specifically, in NAFLD, DAMPs released from damaged hepatocytes bind to DAMP receptors on liver macrophages and hematopoietic stem cells, leading to the activation of KCs, which then produce chemokines and cytokines such as TNF-α, IL-1β, and CCL2 (Zwicker et al., 2021; Rajak, 2024). KCs also play a crucial role in the fibrotic stage of NAFLD. They secrete TGF-β and other pro-fibrotic factors, activate HSCs, and promote extracellular matrix deposition and progression of liver fibrosis (Carter and Friedman, 2022; Wallace et al., 2022). The figure below illustrates the plasticity of KCs in the liver and how they are influenced by other liver cells (Figure 3).

The influence of different local microenvironments allows KCs to be activated into distinct phenotypes, which are primarily classified as classical activation (M1) and alternative activation (M2). KCs with an M1 phenotype secrete pro-inflammatory cytokines, present antigens, and actively regulate immune responses. In contrast, KCs with an M2 phenotype primarily contribute to the secretion of anti-inflammatory factors and negatively regulate immune reactions (Muntjewerff et al., 2020).

### 4.1 M1 macrophages

M1 macrophages, also known as classically activated macrophages, play a pivotal role in the pathogenesis of NAFLD and NASH. The polarization towards the M1 phenotype is of particular significance. Activated M1 macrophages can release a plethora of pro-inflammatory factors including TNF-α, IL-1β, IL-6, IL-12, IL-18, and IL-23, as well as type I interferons and a multitude of CXCL 1, CXCL3, and CXCL5. These factors induce the activation of Type 1 Helper T Cell (Th-1) responses and promote complement-mediated phagocytosis and type I inflammation, exerting pro-inflammatory effects and impeding tissue regeneration ultimately (Sutti et al., 2014). Furthermore, M1-polarized macrophages also contribute to the development of NAFLD and NASH by participating in oxidative stress responses and lipid metabolism abnormalities (Seth et al., 2015; Zhang et al., 2019; Li X.-Y. et al., 2022).

The interaction of the “gut-liver axis” can be exacerbated by LPS and inflammatory mediators, which can lead to the development of hepatic diseases (Odegaard et al., 2008; Yunna et al., 2020). Pathogens bind to KCs under the influence of LPS, leading to the production of proinflammatory cytokines including IL-6, IL-12, and IL-18. This process causes liver injury and inflammation (Casari et al., 2023).

### 4.2 M2 macrophages

In contrast, M2 macrophages are induced by Th2 cytokines and are referred to as “alternative” M2 macrophages. Following the initial polarization of macrophages to the M1 phenotype to resist pathogen invasion, these cells undergo a second polarization and participate in the anti-inflammatory response of the M2 phenotype.

The activation of the “alternative” M2 macrophages has been demonstrated to ameliorate hepatic steatosis and insulin resistance (Odegaard et al., 2008). M2 macrophages are capable of clearing tissue debris and apoptotic cells, as well as promoting angiogenesis and fibrosis. They also control the development of inflammation, repair damaged tissue, and boost insulin sensitivity (Yunna et al., 2020; Casari et al., 2023).

Upon activation by Th2 cytokines such as IL-4 and IL-13, M2 macrophages produce anti-inflammatory cytokines, including IL-10 and TGF-β, to help suppress inflammatory responses, inhibit fibrosis, and promote tissue repair (Bouhlel et al., 2007; Casari et al., 2023). Based on the activation markers they express, M2 macrophages are typically divided into three subgroups in response to a variety of stimulating factors: M2a macrophages are induced by IL-4 or IL-13, M2b macrophages are induced by Fc receptors and immune complexes, and M2c macrophages are triggered by IL-10, TGF-β, and glucocorticoids (Odegaard et al., 2008; Friedman et al., 2018). Each subtype is involved in the inflammatory response and the promotion of tissue repair and injury. M2a is engaged in anti-inflammatory processes, repair, inflammation inhibition, tissue repair, and the promotion of fibrosis. M2c may regulate inflammatory responses and inhibit fibrosis processes (Muntjewerff et al., 2020). Previous studies have revealed that the expression levels of M2-related markers are elevated in MASH patients, suggesting that these hepatic macrophages may participate in liver remodeling after hepatocellular injury by promoting tissue repair. However, this also increases the risk of fibrosis development (Rensen et al., 2009). This appears to be at odds with the anti-inflammatory and cell-repairing functions typically associated with M2 macrophages. Interestingly, M2c macrophages can secrete numerous cytokines, chemokines, and proteases, which promote tumor angiogenesis, growth, metastasis, and immune suppression, thereby contributing to hepatic fibrosis (Kwon et al., 2020).

In addition to the conventional classifications previously mentioned, ongoing research continues to unveil novel subpopulations of macrophages. These subpopulations may exhibit functional characteristics that fall within the M1-M2 spectrum or display significant phenotypic variations compared to previously identified groups. For example, recent discoveries have identified a novel subpopulation of macrophages, Trem2+ lipid-associated macrophages, which were first described in adipose tissue. In the context of NAFLD, lipid-associated macrophages are known to reside in fibrotic regions, macrophage clusters, or hepatic crown-like structures. It is noteworthy that they have been demonstrated to ameliorate hepatic fibrosis and limit NASH (Hendrikx et al., 2022). Additionally, TREM2 is a single-pass transmembrane receptor belonging to the immunoglobulin superfamily. It was discovered that TREM2 was almost undetectable in KCs, but predominantly expressed in MoMfs. The expression of TREM2 was induced by the release of sphingosine-1 phosphate by apoptotic hepatocytes, and its high expression ensured that macrophages could effectively and promptly clear apoptotic hepatocytes (Wang X. et al., 2023).

Ubiquitin-specific protease 19 (USP19) is a deubiquitinating enzyme that is anchored in the endoplasmic reticulum (ER) and plays a role in regulating ER-associated protein degradation and DNA damage repair. It was observed to inhibit NLRP3 inflammasome activation by increasing autophagy flux and decreasing the generation of mitochondrial reactive oxygen species. Additionally, USP19 demonstrated the capacity to inhibit the proteasomal degradation of inflammasome-independent NLRP3 by cleaving its polyubiquitin chains, thereby acting as an anti-inflammatory switch that inhibits inflammatory responses while promoting M2-like macrophage polarization (Liu T. et al., 2021).

### 4.3 The shift from M1 to M2 polarization of hepatic macrophages in NAFLD

Macrophages can undergo functional changes in response to the local cytokine environment. These changes are reversible and can be influenced by appropriate stimuli, leading to macrophage plasticity and reprogramming (Lee et al., 2022; Ni Y. et al., 2022).

To limit tissue inflammation and maintain internal homeostasis, it is essential to utilize effective anti-inflammatory mechanisms. Macrophages induce inflammatory responses and release pro-inflammatory mediators including cytokines, chemokines, and reactive oxygen and nitrogen intermediates during the acute phase of inflammation when they initially polarize into classical M1 types. This process activates multiple anti-microbial pathways that aid in the clearance of causative agents and the resolution of inflammation. However, to prevent tissue damage to the host, it is essential to control this response. The polarization of M2 macrophages is essential for the proper initiation, development, and elimination of inflammation. M2 macrophages produce anti-inflammatory cytokines, mediators, cytokines, and chemokines that modulate M1 macrophages, attenuate the inflammation response, and promote and expedite the process of wound repair and tissue regeneration. The equilibrium and polarization status of M1 and M2 macrophages are critical for the proper initiation, development, and elimination of inflammation (Chen et al., 2021).

Previous research has demonstrated the significant role of the transition between M1 and M2 polarization of hepatic macrophages in NAFLD (Ni Y. et al., 2022). This transition is linked to a reduction in M1 indicators and a rise in M2 indicators. Research has shown that switching liver macrophages from M1 to M2 polarization can help reduce inflammation and promote tissue repair in NAFLD. This is associated with a decrease in the secretion of pro-inflammatory cytokines and an increase in anti-inflammatory and reparative factors. This ultimately influences the progression and outcome of NAFLD (Fan et al., 2022; Lee et al., 2022; Pant et al., 2023).

Research conducted on NAFLD mice has highlighted the involvement of the long non-coding RNA SNHG20 in NAFLD advancement. Inhibiting SNHG20 hindered KCs polarization, thus preventing NAFLD. RNA silencing resulted in a reduction in M1 polarization, whereas overexpression led to M2 polarization. Overexpression of SNHG20 was found to activate STAT6, a key player in promoting M2 macrophages. These findings suggest that the progression from NAFLD to hepatocellular carcinoma (HCC) is reliant on this STAT6 activation (Wang B. et al., 2019).

Hepatic resident macrophages may play an important role in the inhibition of inflammation and fibrosis associated with NAFLD through the expression of peroxisome proliferator-activated receptor-γ (PPAR-γ). PPAR-γ is involved in regulating various processes related to NAFLD, including hepatic steatosis, inflammation, insulin resistance, and fibrosis. With potent anti-inflammatory properties, PPAR-γ has been identified as a crucial regulator of M2 polarization in previous studies (Chinetti et al., 2003; Bouhlel et al., 2007). In NAFLD, there is a disruption of PPAR-γ activity, which results in impaired lipid metabolism and increased inflammation in the liver (Wang Y. et al., 2020). Wenjing Luo has proposed further study of the specific role of PPAR-γ and the relationship between PPAR-γ and the transition from M1 to M2 polarization. The upregulated expression of PPAR-γ can shift lipid-induced macrophage polarization from M1 to M2 phenotype (Luo et al., 2017).

In NAFLD model mice, macrophage-specific PPAR-γ deficiency leads to enhanced metabolic activation of macrophages, which promote the migration and activation of HSCs through the secretion of IL-1β and CCL2, thereby exacerbating the progression of NASH (Ni X. X. et al., 2022). In PPAR-γ-deficient NASH livers, monocyte-derived macrophages became the predominant macrophage type, accompanied by strong activation of HSCs and exacerbated NASH. These findings indicate that by regulating PPAR-γ in macrophages to balance the M1/M2 phenotype of metabolically activated macrophages, thus affecting their interaction with HSCs, it may influence hepatic oxidative stress, steatosis, inflammation, and fibrosis during NAFLD progression (Kratz et al., 2014). Therefore, targeting PPAR-γ in macrophages may offer a promising strategy for treating NAFLD. One potential avenue for intervention is to regulate PPAR-γ activity to restrain M1 polarization. This approach has the potential to halt the progression of NAFLD and subsequently repair hepatic cells from inflammatory injury. For instance, PPAR-γ agonists, such as thiazolidinediones, have been identified as promising therapeutic agents for NAFLD. PPAR-γ agonists have been shown to activate and improve lipid metabolism and insulin sensitivity, as well as reduce inflammation in the liver. However, the use of these drugs to treat NAFLD remains controversial, and further research will be needed to determine their long-term safety.

To gain a deeper understanding of the activation process of hepatic macrophages, we have gathered information on several biomarkers. These biomarkers are utilized to predict the activation status and polarization forms of macrophages (Table 1).

**TABLE 1:** Biomarkers that predict activation of KCs in NAFLD and NASH.

Polarization type	Activator	Biomarkers	Effect	Reference
M1	LPS, GM-CSF, IFNG	TNF-α, IL-1, IL-6, iNOS↑	Promoting M1 macrophages activation, exacerbating the inflammatory response, and enhancing hepatocyte injury	Nagashimada and Honda (2021), Chen et al. (2023)
M2a	IL-4, IL-13	IL-10, TGF-β, CCL17, CCL18, CCL22↑	Promoting cell growth, tissue repair, and endocytosis	Wang et al. (2023b)
M2b	immune complex, IL-1β	TNF-α, IL-1β, IL-6, IL-10↑	Regulating immunity and response	Chen et al. (2023)
M2c	Glucocorticoid, IL-10	TGF-β, CCL16, CCL18, MerTK↑	Promoting phagocytosis of apoptotic cells and fibrosis progression	Pastore et al. (2019)

## 5 The macrophage autophagy in NAFLD

Autophagy represents a critical protein degradation system that is mediated by lysosomes (Xu J. et al., 2023). The role of autophagy in the development of NAFLD and NASH is complex. In the initial stages of NAFLD, autophagy is frequently elevated as a defensive mechanism to eliminate surplus lipids and damaged cellular components (Yang Y. et al., 2022). Autophagy plays a pivotal role in the degradation of lipid droplets, which is vital for preventing the excessive accumulation of lipids, a defining feature of NAFLD (Robichaud et al., 2021; Yang et al., 2023). Autophagy also facilitates the clearance of damaged mitochondria, thereby reducing the production of reactive oxygen species and consequently alleviating oxidative stress. The accumulation of reactive oxygen species is a consequence of autophagy dysfunction, which in turn exacerbates oxidative stress (Liu et al., 2020). As NAFLD progresses, autophagic synthesis and degradation are initially activated but then gradually inhibited. This indicates that while the formation of autophagosomes is increased, the subsequent degradation process may be impeded (Jin et al., 2023). Autophagy plays a role in the clearance of damaged organelles and inflammatory mediators, thereby reducing the level of inflammation. The dysfunction of autophagy has been demonstrated to result in the exacerbation of inflammation, which is a pivotal factor in the pathogenesis of NASH (Wang X. et al., 2019). In the advanced stages of NASH, autophagy is typically inhibited. This can lead to the accumulation of damaged organelles and proteins, further exacerbating oxidative stress and inflammation. Chaperone-mediated autophagy is a form of autophagy that selectively delivers soluble proteins for lysosomal degradation. MoMfs were the dominant macrophage population in NASH, and the function of chaperone-mediated autophagy was found to be impaired in these cells. Nup85 is a cytoplasmic protein that interacts with CCR2. Macrophage chaperone-mediated autophagy dysfunction induced the inhibition of Nup85 degradation, promoting liver inflammation and disease progression of NASH (Zhang M. et al., 2023). The latest research has revealed that a high-fat diet induces oxidative stress, which in turn activates the STING and YAP pathways in hepatic macrophages. STING interacts with YAP to regulate the expression of the TMEM205 gene, which affects the autophagic degradation of lipid droplets. Consequently, activation of the STING-YAP axis can promote lipid degradation (Yang et al., 2023).

## 6 Discussion

In the ever-evolving landscape of NAFLD and NASH, the role of hepatic macrophages has emerged as a pioneering field of study with far-reaching implications. This review has illuminated the remarkable versatility of hepatic macrophages in modulating the intricate dance of inflammation, fibrosis, and metabolic perturbations within the liver. The innovative discoveries we’ve explored here underscore several key takeaways. Firstly, the complexity of NAFLD and NASH stems from their diverse manifestations and the multitude of pathophysiological metabolic and inflammatory processes involved. This complexity is further reflected in the heterogeneously regulated populations of macrophages found in the liver. These macrophage populations coexist at various stages of liver injury, either promoting or inhibiting disease progression. However, the interplay between these hepatic macrophage populations and their communication with other cells in the liver environment remains inadequately understood. Secondly, hepatic macrophages, whether they be the resident KCs or infiltrating MoMFs, exhibit remarkable plasticity. Their ability to transition between M1 and M2 phenotypes based on local cues presents a remarkable opportunity for therapeutic interventions aimed at fine-tuning their activities. Thirdly, we elucidate the dynamic interactions among MoMFs, HSCs, T cells, and liver sinusoidal endothelial cells, expanding our understanding of the immune pathogenesis of NAFLD and NASH, and providing potential avenues for therapeutic intervention targeting immune dysregulation and hepatic inflammation.

Additionally, various natural products and drugs have been explored as potential therapeutic agents targeting KCs or macrophages to treat NAFLD, including CCR2/CCR5 antagonists and galectin-3 inhibitors. However, the majority of these interventions are currently limited to *in vivo* animal studies, which highlights the necessity for additional clinical trials to assess their efficacy and safety in treating NAFLD patients. Fortunately, recent advancements in biomedical research techniques offer opportunities to reclassify macrophage subsets based on their transcriptomic and proteomic profiles, spatial organization, dynamic behavior, and functional capacity in different disease stages (Han H. et al., 2023). Moreover, the integration of knowledge from extrahepatic signals (such as those from the gut and adipose tissue) and patient-specific factors (including nutrition, genetics, and comorbidities) is facilitated by progress in bioinformatics and data science. This integration holds promise for tailoring therapeutic strategies to modify macrophages in NAFLD progression. The enigma of NAFLD and NASH, intricately woven into the cellular dialogues of the liver, continues to be unraveled.
